# Evaluation of cell proliferation, apoptosis, and dna-repair genes as potential biomarkers for ethanol-induced cns alterations

**DOI:** 10.1186/1471-2202-13-128

**Published:** 2012-10-25

**Authors:** Steven D Hicks, Lambert Lewis, Julie Ritchie, Patrick Burke, Ynesse Abdul-Malak, Nyssa Adackapara, Kelly Canfield, Erik Shwarts, Karen Gentile, Zsuzsa Szombathyne Meszaros, Frank A Middleton

**Affiliations:** 1 Departments of Neuroscience & Physiology, Upstate Medical University, 750 East Adams Street, Syracuse, NY, 13210, USA; 2 Department of Psychiatry, Upstate Medical University, Syracuse, NY, USA; 3 Developmental Exposure Alcohol Research Center, Binghamton University, Binghamton, NY, USA

**Keywords:** Biomarker, Peripheral blood, Microarray, p53, DNA damage, Alcoholism, Neuroimaging

## Abstract

**Background:**

Alcohol use disorders (AUDs) lead to alterations in central nervous system (CNS) architecture along with impaired learning and memory. Previous work from our group and that of others suggests that one mechanism underlying these changes is alteration of cell proliferation, apoptosis, and DNA-repair in neural stem cells (NSCs) produced as a consequence of ethanol-induced effects on the expression of genes related to p53-signaling. This study tests the hypothesis that changes in the expression of p53-signaling genes represent biomarkers of ethanol abuse which can be identified in the peripheral blood of rat drinking models and human AUD subjects and posits that specific changes may be correlated with differences in neuropsychological measures and CNS structure.

**Results:**

Remarkably, microarray analysis of 350 genes related to p53-signaling in peripheral blood leukocytes (PBLs) of binge-drinking rats revealed 190 genes that were significantly altered after correcting for multiple testing. Moreover, 40 of these genes overlapped with those that we had previously observed to be changed in ethanol-exposed mouse NSCs. Expression changes in nine of these genes were tested for independent confirmation by a custom QuantiGene Plex (QGP) assay for a subset of p53-signaling genes, where a consistent trend for decreased expression of mitosis-related genes was observed. One mitosis-related gene (*Pttg1*) was also changed in human lymphoblasts cultured with ethanol. In PBLs of human AUD subjects seven p53-signaling genes were changed compared with non-drinking controls. Correlation and principal components analysis were then used to identify significant relationships between the expression of these seven genes and a set of medical, demographic, neuropsychological and neuroimaging measures that distinguished AUD and control subjects. Two genes (*Ercc1* and *Mcm5*) showed a highly significant correlation with AUD-induced decreases in the volume of the left parietal supramarginal gyrus and neuropsychological measures.

**Conclusions:**

These results demonstrate that alcohol-induced changes in genes related to proliferation, apoptosis, and DNA-repair are observable in the peripheral blood and may serve as a useful biomarker for CNS structural damage and functional performance deficits in human AUD subjects.

## Background

Alcohol use disorders (AUDs), which encompass both alcohol addiction and dependence, cause significant structural and functional deficits in the adult human brain. Adult alcoholics display neuronal loss in the hippocampus and superior frontal association cortex [1,2] along with increased ventricle size and sulcal widening [3]. Cognitive deficits often accompany these changes and are characterized by impaired anterograde spatial memory [4] and episodic learning [5]. Yet, the predominant clinical tests used to identify pathology in AUD subjects focus on molecular changes within the liver [6,7].

Alcohol exposure also causes molecular changes within the central nervous system (CNS). For example, long-term exposure to alcohol has been associated with changes in gene expression related to neurogenesis, neuroprotection and neurodegeneration within the frontal cortex [8,9], cell cycle and neuronal differentiation within the superior frontal cortex [10], and DNA-repair and apoptosis within the nucleus accumbens and prefrontal cortex [11]. Moreover, some of the reproducible expression changes in these classes of genes were found to reliably predict AUD status [9]. Despite these compelling results based on post-mortem human tissue, there are numerous limitations to such studies that reduce their potential utility for biomarker discovery in AUDs, including CNS tissue availability, agonal state, post-mortem interval, brain pH and other variables.

Studying alterations in gene expression levels in peripheral blood offers an intriguing alternative to brain-based biomarkers. It is believed that 80% of genes expressed in brain tissue are expressed in blood, and the subtle changes in the brain associated with injury or disease may be reflected by changes in gene expression in blood [12]. The common origin of microglial cells and white blood cells from bone marrow-derived monocytes allow them to share the expression of many surface receptors and signaling proteins related to the immune response [13]. It has been posited that peripheral lymphocytes recruited through the blood brain barrier in response to brain injury may be exposed to previously hidden antigens, which could lead to clonal expansion and differentiation of lymphocytes as well as changes in circulating lymphocyte gene expression profiles [14]. In support of this, studies have demonstrated overlap in the expression of Alzheimer’s-related genes in the blood and brain suggesting the potential for particular genes in peripheral blood cells to function as biomarkers for diagnosis and prognosis and also as targets for therapy of CNS disease and injury [13].

Previously we have demonstrated that the exposure of cultured NS-5 neural stem cells (NSCs) to alcohol results in significant changes in the expression of genes related to cell proliferation, DNA repair, and apoptosis – functions related to p53 signaling [15,16]. The present study proposes to examine whether changes in these pathways are reliably and robustly observed in the peripheral blood of animal drinking models and human AUD subjects. Because NSCs and peripheral leukocytes are both highly proliferative cell populations, we hypothesize that alcohol-induced changes in p53 signaling genes will be similar in these cell types. Furthermore, we hypothesize that some of the consistent changes in human AUD subjects will be correlated with structural and functional measures of CNS function as well as their alcohol use severity.

## Methods

### Mouse neural stem cells

An overview of the experimental design and workflow is provided in Additional file 1 figure S1. The present study specifically examined human and rat PBLs for changes in expression of genes related to p53 signaling, cell proliferation, apoptosis and DNA repair. This derived from our previously published microarray-based examination of changes in mouse NSCs exposed to ethanol *in vitro*[15], where robust expression alterations in these pathways were observed and confirmed using both real-time quantitative RT-PCR and immunocytofluorescent staining. The original microarray data set on the mouse NSCs is available for download in the NCBI Gene Expression Omnibus (GSE 19436). Because the methods have also been described in detail [15,16], they are only summarized here. Briefly, a line of non-immortalized, pluripotent NS-5 NSCs was derived from embryonic mouse brains at day 11.5 of gestation. Approximately 100,000 cells were plated (in an 8 ml volume) in 100 mm petri dishes coated with poly-L-ornithine hydrobromide (15 ug/ml; Sigma) and laminin (10 ug/ml; Sigma). Cultures were maintained in supplemented Euromed-N medium until they reached 30% confluency and then exposed for 48 hours to either FGF2 or TGFβ1 (10 ng/ml) or ethanol (400 mg/dL) and a growth factor in Euromed Media (Peprotech). Three petri dishes were used for each condition and placed inside airtight containers with 400 mg/dL ethanol or sterile water to maintain the ethanol concentration of the media. Total RNA was purified from each sample and used for either microarray analysis using the GeneChip Mouse 430 2.0 Array (Affymetrix) or real-time quantitative RT-PCR using the RT^2^ First-Strand Kit and PCR arrays (SA Biosciences).

### Rat studies

A total of 20 (10 male and 10 female) Long-Evans rats obtained from Harlan Labs were used for these studies. Animals were cared for in accordance with protocols approved by the Committee for Human Use of Animals (CHUA) at SUNY Upstate Medical University. These animals were housed in individual cages and exposed to a 12 hour reverse light/dark cycle. Fresh food was provided to all rats at 10:00 AM. At postnatal day 29 rats were split into four groups (two treatment and two control). One group (n = 10; 5 male, 5 female) of rats was weaned onto an *ad lib* liquid ethanol-containing diet for 3 weeks. Rats began the ethanol (ET) diet at 2.2% v/v and were gradually weaned up to 4.5% v/v and finally 6.7% v/v. After this initial exposure, rats received 6.7% v/v liquid ethanol diet for three consecutive days each week, followed by 4 days of *ad lib* solid rat chow pellets (Purina). A second group of rats (n = 10) was paired with the ET-fed rats based on gender and initial body weight (pair-fed control rats), and received aliquots of a liquid control diet defined by the amount of food consumed by the paired ET-fed rats.

The liquid diet was obtained from OpenSource Research Diets™. Maltose replaced ethanol in the control pair-fed (PF) diet to match for caloric and nutritional content. Throughout the study duration, regular records of the rats’ body weights and ethanol (or control diet) consumption were maintained. At the end of the study, all rats in each group were euthanized with CO_2_ and samples of their cardiac blood were obtained for RNA isolation into PaxGene RNA stablization solution or serum separator tubes for liver enzyme testing (AST, ALT, ALP, total protein). Rats were then transcardially perfused with approximately 30 mL Phosphate Buffered Saline (PBS) followed by 30 mL RNAlater® (Qiagen) which was used to stabilize RNA in the brain tissues and was not collected. RNA was extracted according to standard PaxGene protocols and differential expression of transcripts was investigated using a Rat Gene 1.0 ST Array (Affymetrix),

RMA-normalized data from the rat and previously-published mouse microarrays were analyzed using Partek Genomic Suite to identify common patterns of expression level changes in a set of 350 genes of interest involved in cell proliferation, apoptosis, DNA-repair, and p53-signaling pathways according to the SA Biosciences database. A pairwise two-way analysis of variance (ANOVA) was used to identify genes altered significantly by alcohol in the rats for comparison with the mouse NS-5 data on the same genes. The mouse and rat data for these genes were then compared using Pearson correlation. Significance for this was based on a step-up multiple testing correction, with the false discovery rate (FDR) set at 0.1. Thirty-four of these genes were selected for validation in rats (and humans) using a QuantiGene Plex 2.0 (QGP) assay (Affymetrix), as described below. The complete set of genes included in the QGP assay is displayed in Table 1.

**Table 1:** Ethanol-induced gene expression changes in human lymphoblasts

**Gene**	**Log2 Change**	***T*test P**	**Mann–Whitney P**
***TP73***	***-0.24***	***0.0076***	***0.0495***
***HUS1***	***-0.33***	***0.0186***	***0.0495***
***GADD45A***	***-0.38***	***0.0272***	***0.0495***
***ATM***	***-0.30***	0.0525	***0.0495***
*CASP3*	-0.31	0.0725	0.1266
***PTTG1***	***-0.11***	0.0764	***0.0495***
*CCND1*	-0.44	0.1024	0.1266
*CDK1*	-0.37	0.1143	0.1266
*CDC25C*	-0.29	0.1196	0.1266
***MUTYH***	***-0.25***	0.1658	***0.0495***
*ERCC1*	-0.19	0.1724	0.1266
***CD40***	***-0.46***	0.1801	***0.0495***
*CCNB2*	-0.12	0.2423	0.5127
*ATR*	-0.23	0.2561	0.1266
*CHEK1*	-0.17	0.2595	0.1266
*CDK4*	0.09	0.3009	0.5127
*E2F3*	-0.34	0.3205	0.2752
*CCNA2*	-0.35	0.3559	0.5127
*CASP8*	-0.20	0.3597	0.2752
*PCNA*	-0.26	0.3729	0.5127
*APEX1*	0.09	0.3942	0.2752
*PARP1*	0.10	0.4639	0.2752
*JUN*	-0.22	0.5296	0.5127
*XRCC5*	0.05	0.5766	0.5127
*FADD*	-0.09	0.5912	0.5127
*FASLG*	-0.37	0.6082	0.8273
*TNFRSF10B*	0.06	0.6242	0.5127
*RACGAP1*	-0.15	0.6435	0.8273
*APAF1*	-0.08	0.8085	0.8273
*MDM4*	-0.06	0.8190	0.5127
*TP53*	-0.07	0.8410	0.8273
*HPRT1*	0.00	0.8822	0.5127
*PPIA*	0.00	0.8917	0.5127
*BCL2*	0.03	0.8928	0.8273
*MCM5*	-0.02	0.9212	0.5127
*MYC*	0.02	0.9242	0.8273

These genes represent the complete set of genes analyzed by QuantiGene Plex 2.0. assays in cultured human lymphoblasts as well as primary human and rat PBLs.

Nominally significant observations are indicated in boldface italics.

### Human studies

#### Recruitment and inclusion/exclusion criteria

All procedures were approved by the Institutional Review Board (IRB) of SUNY Upstate Medical University as well as Crouse Irving Memorial Hospital. A total of 65 subjects participated in this study. This included 50 subjects with a current diagnosis of an alcohol use disorder (AUD) and 15 non-drinking control subjects. The AUD subjects included 40 with alcohol dependence (AD) and 10 with alcohol abuse (AA), according to DSM-IV criteria. Subjects were recruited from the greater Syracuse area, the Crouse Irving Memorial Hospital Chemical Dependence Treatment Services Clinic, Syracuse Behavioral Health, Tully Hill Chemical Dependency Treatment Center, ARISE Child and Family Services, and Syracuse University.

Exclusion criteria included age less than 18 years, or greater than 60 years, weight greater than 270 lbs., pregnancy, co-morbid drug abuse (except for cigarette smoking), history of head injury with loss of consciousness or co-morbid medical conditions including diabetes, cancer, hepatitis C, neurological diseases (e.g. seizure disorder) and major mental illness (except for anxiety disorders and major depression). In addition patients with metallic implants of any kind (braces, pacemakers, etc.) or claustrophobia were excluded based upon MRI incompatibility. The Structured Clinical Interview (SCID) for the Diagnostic and Statistical Manual of the American Psychiatric Association, 4^th^ edition (DSM-IV) was administered to all subjects by a psychiatrist to establish a definitive diagnosis of alcohol dependence or abuse. As a supplement to the SCID, the Semi-Structured Assessment for the Genetics of Alcoholism Version IV (SSAGA-IV) was administered to obtain participant demographics, past medical history, and tobacco and alcohol use in a format that could be easily cross-referenced to other studies.

#### Medical, demographic, and neuropsychological assessments

After obtaining informed consent, subjects were initially screened for drugs of abuse using a dipstick urine toxicology screen and breath alcohol test. Over the course of two subsequent appointments, subjects completed a blood draw, brief neurological exam, neuropsychological testing, and a structural brain MRI. A total of three blood tubes were collected from each subject: one for RNA isolation (PaxGene) from leukocytes in the venous blood, a second for complete blood cell count and differential white blood cell count and the third for routine laboratory measures that determined basic metabolic status, including blood glucose, and serum levels of AST, ALT, and GGT to reveal liver damage. The total RNA was extracted from the samples of venous blood according to PaxGene protocol and processed by the SUNY Microarray Core facility (SUNYMAC) to determine the expression level of a set of 34 genes using a custom-designed Quantigene Plex 2.0 assay (Affymetrix), as described below.

The neurological examination screened for obvious signs of cerebellar damage (ataxia, dysmetria, decomposition of movement, dysdiadochokinesia, and smooth pursuit eye movement impairments). Neuropsychological tests included the Wechsler Abbreviated Scale of Intelligence (WASI), the Wechsler Memory Scale (WMS) and selected scales from the Delis-Kaplan Executive Function System (DKEFS), including the trail-making tasks and verbal fluency tasks. These tests were chosen based upon their extensive use in clinical practice for assessment of cognition.

The structural brain MRI series was obtained from all human subjects in the sagittal plane on a 1.5 T Philips Gyroscan scanner from Philips Medical Systems™. Each scan utilized the following T-1 weighted inversion recovery 3-D pulse sequence: TE = 4.6; TR = 20; 2 repetitions, matrix size 256 X 154; FOV 24; multishot = 32; TFE pre IT shortest (394 ms), 1.5 mm slice thickness. Analysis of the images was performed to determine the extent of CNS damage (if any) in AUD subjects compared to non-drinking controls. Images were evaluated for evidence of gross structural abnormalities by a trained neuroanatomist and any unusual findings were followed up with a neuroradiologist. The analysis that was performed utilized a semi-automated image reconstruction and segmentation software suite (FreeSurfer) developed by the Martinos Center (Harvard/MGH). This software generated parcellated volumes for more than 360 cortical and subcortical brain structures, including cortical gray matter, white matter, gyri and sulci.

Analysis of the combined medical, demographic, neuropsychological and neuroimaging data was performed to identify significant group differences between AUD and control subjects. A total of 423 variables were tested, with Benjamini-Hochberg FDR-corrected significance set at 0.1. Significant variables from this analysis were combined with significantly affected genes identified in the QuantiGene Plex 2.0 analysis (described below) and subjected to correlation analysis and principal components analysis (PCA). Genes were selected for correlation and PCA analysis if they displayed nominally significant changes in the QuantiGene Plex assay (p ≤ 0.05) as well as nominally-significant changes in either human lymphoblasts exposed to ethanol, mouse NS-5 cells exposed to ethanol, or PBLs from binge-drinking adolescent rats. For the correlation analysis in human subjects, we used Pearson r values, with r to t transformation used to determine nominal p values, which were then adjusted using a Bonferroni correction (based on examination of 3039 unique correlation tests). For the PCA analysis, we focused on the factors that described 75% of the variance of the significantly changed variables and performed hierarchical clustering on the oblique factor weights. Genes of interest identified in this approach were subsequently examined for significant differences between AA, AD and Control subjects using a one-way analysis of variance (ANOVA) with a Fisher’s protected least significant difference test used for post-hoc comparisons.

#### Lymphoblast assays

To complement the human, rat and mouse studies, we also examined cultured lymphoblasts (LBs) for ethanol-induced changes in gene expression. Cells for these studies were obtained from an immortalized LB cell line that had been derived from Epstein-Barr Virus transformed B lymphocytes of a healthy non-alcoholic control subject. Frozen stocks of these cells were thawed and initially grown in RPMI 1640 Complete media in T-75 flasks, with 10% heat-inactivated fetal bovine serum, penicillin-streptomycin (100 U), gentamicin (100 ug/ml), L-glutamine (2 mM), and HEPES (10 mM). Cells were split after reaching approximately 75% confluency. Approximately 50,000 cells were then plated (in a 6.5 ml volume) into 4 replicate wells of a 6 well plate that had been coated with laminin (10 ug/ml; Sigma) for treatment with either ethanol (200 mg/dL) or control media. The concentration of ethanol in the media was measured at regular intervals throughout each exposure using a GM-7 Microstat (Analox Instruments). At the end of the treatments, RNA was isolated from each well and used for quantification of gene expression using the QuantiGene Plex 2.0 Assay, as described below.

### QuantiGene plex 2.0 Assays

Rat and human total RNA samples were processed according to the Affymetrix QuantiGene Plex 2.0 Assay Manual. Briefly, a working bead mix was prepared containing lysis mixture, blocking reagent, capture beads, and 2.0 probe set. The bead mix was dispensed into the hybridization plate, and 20ul of total RNA was added to each well. For background control wells, 20ul of sterile nuclease-free water was added to the bead mix. The hybridization plate was sealed with a pressure seal and placed into a VorTemp 56 shaking incubator (Fisher). The plate was incubated for 22 hours at 54 °C and 600RPM.

After hybridization, the wash solution, pre-amplifier, amplifier, label probe, and streptavidin-phycoerythrin (SAPE) solutions were prepared according to the manual instructions. The hybridized samples were then transferred from the hybridization plate to the magnetic separation plate. Samples were washed using the Affymetrix Hand-Held Magnetic Plate Washer, and incubated sequentially for 1 hour each (50 °C at 600RPM) with the pre-made amplifier solutions (pre-amplifier, amplifier, and label probe). The SAPE solution was then added to the plate and samples were incubated at room temperature for 30 minutes at 600RPM. The plate was covered in foil to protect samples from light. The unbound SAPE was then washed away using the SAPE Wash Buffer from the QuantiGene Plex Kit. Then, 130ul of SAPE Wash Buffer was added to each sample, and the plate was shaken at room temperature for 3 minutes at 800RPM to resuspend the beads. The plate was read immediately using the BioRad BioPlex 200 instrument. The settings on the BioPlex instrument were set to: sample size 100ul; timeout 60 seconds; Bead Events/Bead region 100.

Fluorescent readings from blank wells were subtracted from fluorescent values for each mRNA of interest. These values were then normalized against the geometric mean expression of two control genes for each sample: cyclophilin A (PPIA) and hypoxanthine-guanine phosphoribosyltransferase (HPRT1). Expression values for each gene were multiplied by a constant and compared between alcohol-exposed and non-exposed samples using a two-tailed *t*-test to identify those transcripts significantly altered by alcohol (p < 0.05).

## Results

In this study, we compared the effects of ethanol exposure on expression of genes involved in p53 signaling, cell proliferation, DNA repair and apoptosis in mouse NSCs *in vitro* with the effects seen in circulating PBLs obtained from binge drinking adolescent rats. The combined results were used to examine changes in the same set of genes in cultures of human lymphoblasts exposed to ethanol *in vitro* and in PBLs obtained from human subjects with AUD. The expression data for the most robustly affected genes in human AUD subjects were also examined for possible associations to various demographic, medical, neuropsychological, and neuroimaging variables.

### Rat drinking data, estimated blood ethanol concentrations, and liver enzymes

All of the binge-drinking and pair-fed control rats gained weight normally through the 3 week time course of the study. Males tended to gain significantly more weight than females (increase = +121% vs +86% of their starting body weight, p < 0.01). The weight gain experienced by the binge drinking rats was not significantly different from their pair-fed controls (increase = +98% vs +108% of their starting body weight, p = 0.07). Binge drinking rats consumed an average of 278.4 ml of 6.7% ethanol-containing liquid diet per Kg of body weight. The amounts consumed by males and females did not differ significantly (p > 0.12). Because the rats included in this study were engaged in binge-drinking, but had not received ethanol on the morning of their euthanasia, it was not possible to directly obtain accurate estimates of their binge state blood ethanol concentrations (BECs). Nonetheless, based on our past experience with the same strain and ages of rats being fed the identical 6.7% ethanol-containing liquid diet, we were able to estimate the approximate peak BECs in our rats based on their age, weight, and daily consumption data using the formula BEC = 0.5 × [Amount consumed per body weight (in ml/Kg)] + 6.76. According to this formula, estimated peak BECs for our rats equaled approximately 145 mg/dl. The estimated values did not differ significantly in the males and females (139.0 vs 150.3, p = 0.125). These values are within the ranges seen in previous studies of adolescent rats fed liquid ethanol diet, and are significantly greater than the 80 mg/dl (0.08%) BECs typically achieved in human binge drinkers who have consumed more than 4 or 5 drinks in a two-hour period (NIAAA.nih.gov).

Data from the liver enzyme panel indicated no changes in serum aspartate aminotransferase (AST) or total protein levels in binge-drinking rats compared with pair-fed controls. However, significant elevations were see in alanine aminotransferase (ALT) levels (66.1 U/L vs 46.4 U/L, p < 0.03) and alkaline phosphatase (ALP) levels (379.6 U/L vs 306.9 U/L, p < 0.005). Both ALT and ALP are sensitive indicators of liver damage. Combined with the estimated BEC values, these data support the notion that the binge drinking paradigm was effectively modeling some aspects of human alcohol abuse.

### Mouse and rat array data comparisons

Our previous microarray analysis of NS-5 cells exposed to ethanol under two different growth factor conditions indicated that 74 out of 350 unique genes involved in p53 signaling, cell proliferation, DNA repair and apoptosis showed significant changes in expression [15]. Of these 74 genes, 14 have been validated (independently confirmed) by qPCR analysis [15,16].

**Figure 1:**
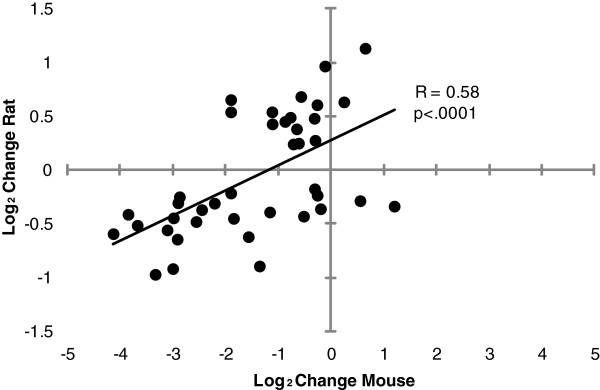
**Comparison of ethanol-induced significant expression changes in cell proliferation, DNA repair, and apoptosis genes seen in mouse NSCs and rat PBLs.** Note that overall, the expression level changes were significantly correlated, particularly for genes with decreased expression in mouse NSCs. Data for the NS-5 comparisons obtained from Hicks et al. study [15].

**Figure 2:**
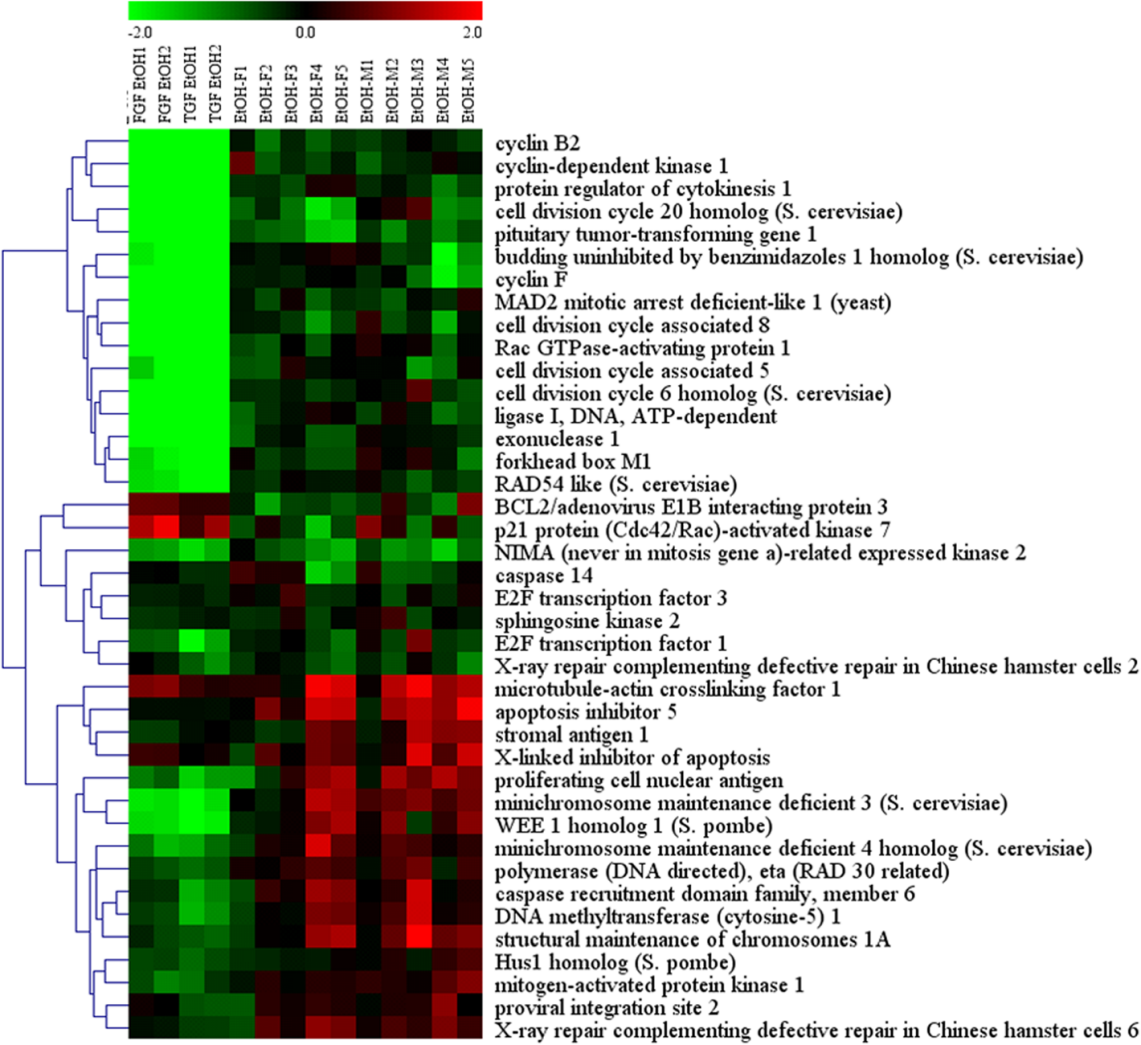
**Hierarchical cluster analysis of ethanol-induced changes in expression of 40 genes involved in cell proliferation, DNA repair, and apoptosis genes seen in mouse NSCs (columns 1-4) and rat PBLs (columns 5-12).** Note that very few differences were noted due to growth factor conditions *in vitro* (FGF, TGF) or to gender. Also note that most of the genes in the top of the cluster diagram are decreased in expression and involved in mitotic cell cycle regulation (e.g., cyclin B2, pituitary tumor-transforming gene 1). In contrast, genes involved in DNA repair and apoptosis tended to show increases in expression, although the changes varied by cell type.

In the present study, using PBLs from a rat adolescent binge drinking model, we found even more striking effects in this set of pathways, with 190 out of 350 unique genes showing significant expression changes after correction for multiple testing. A total of 40 unique genes were significantly affected in both the mouse NSCs and rat PBL studies (Table 2). A direct comparison of the magnitude and direction of expression changes in the two experiments was performed using both correlation analysis and hierarchical clustering. These comparisons revealed that overall the changes observed for the 40 overlapping genes were significantly correlated, particularly for genes with decreased expression in mouse NSCs (Figure 1). Moreover, we also observed very little influence of growth factor conditions *in vitro* (FGF, TGF) or gender on the magnitude and direction of expression change for these genes (Figure 2). Most of the genes with consistently decreased expression were involved in mitotic cell cycle regulation (e.g., cyclin B2, pituitary tumor-transforming gene 1). In contrast, genes involved in DNA repair and apoptosis tended to show increases in expression, although the changes varied somewhat by cell type.

**Table 2:** 40 genes significantly changed in mouse NS-5 cells and rat PBLs

	**Log2 Changes**		**Confirmed Change?**	
**Gene**	**Mouse NS-5**	**Rat PBL**	**Mouse NS-5**	**Rat PBL**
*Api5*	-0.11	0.96		
*Bnip3*	0.56	-0.29		
*Bub1*	-1.56	-0.63	X	
*Card6*	-0.76	0.49	X	
*Casp14*	-0.19	-0.37		
*Ccnb2*	-4.12	-0.60	X	X
*Ccnf*	-2.90	-0.65	X	
*Cdc20*	-3.32	-0.98	X	
*Cdc6*	-2.55	-0.49		
*Cdca5*	-2.89	-0.31	X	
*Cdca8*	-3.10	-0.56		
*Cdk1*	-3.66	-0.52		X
*Dnmt1*	-0.86	0.45		
*E2f1*	-1.15	-0.40		
*E2f3*	-0.25	-0.24		
*Exo1*	-1.84	-0.46		
*Foxm1*	-2.20	-0.31	X	
*Hus1*	-0.60	0.24		
*Lig1*	-2.44	-0.37		
*Macf1*	0.66	1.13		
*Mad2l1*	-2.86	-0.25		
*Mapk1*	-0.70	0.24		
*Mcm3*	-1.89	0.65		
*Mcm4*	-1.10	0.42		
*Nek2*	-1.35	-0.90		
*Pak7*	1.21	-0.34		
*Pcna*	-1.11	0.54		
*Pim2*	-0.29	0.27		
*Polh*	-0.64	0.38		
*Prc1*	-3.84	-0.42		
*Pttg1*	-2.99	-0.92	X	X
*Racgap1*	-2.98	-0.45		X
*Rad54l*	-1.89	-0.22		
*Smc1a*	-0.56	0.68		
*Sphk2*	-0.30	-0.18		
*Stag1*	-0.25	0.60		
*Wee1*	-1.89	0.53		
*Xiap*	0.26	0.63		
*Xrcc2*	-0.51	-0.43		
*Xrcc6*	-0.31	0.48		

These genes were significantly changed by microarray in both mouse NSCs and rat PBLs.

X’s indicate those genes which were independently confirmed using either real-time qRT-PCR (mouse) in the Hicks et al. reports [15,16] or QuantiGene Plex (rat) in the current study.

Based on the correlated changes in the microarray data for the mouse and rat samples, we designed a custom QuantiGene Plex 2.0 assay to enable us to independent confirm the gene expression changes in the rat and determine whether changes in the same class of genes occur in human PBLs from AUD subjects or human lymphoblasts exposed to ethanol. Approximately half (14) of the genes in the custom assay had been previously observed to change in the mouse NSC studies, while 19 were observed to change in the present rat PBL study. A total of 9/19 of these rat PBL genes were independently confirmed to be significantly (nominal p < 0.05) changed in the same direction seen by microarray in the QuantiGene Plex 2.0 assay (*Apaf1*, *Apex1*, *Atm*, *Ccnb2*, *Cdc2/Cdk1*, *Gadd45a*, *Myc*, *Pttg1*, and *Racgap1*). Of these 9 genes, 4 were also significantly changed in the mouse NSC array data (*Ccnb2*, *Cdk1*, *Pttg1*, and *Racgap1*; Table 2).

### Human lymphoblasts

Of the 34 genes examined for expression changes in the human lymphoblasts by the QuantiGene Plex assay, we observed nominally significant changes in a total of 7, according to parametric or non-parametric tests (*Tp73*, *Hus1*, *Gad45a*, *Atm*, *Pttg1*, *Mutyh*, *Cd40*; Table 1). All 7 of these genes were decreased in expression, and one (*Pttg1*) overlapped with the independently confirmed changes seen in the mouse NSCs and rat PBLs (Table 2).

### Human studies

#### Demographic and medical findings

There were no significant differences between our subject groups based on age or gender composition (Table 3). AUD subjects, however, did show significantly reduced gross personal income and years of education, in addition to increased systolic blood pressure (Table 4).

**Table 3:** Demographic and drinking variables of human subjects

	**AUD (n = 50)**	**AD (n = 40)**	**AA (n = 10)**	**Controls (n = 15)**
**Gender (Females, Males)**	**18 F, 32 M**	**13 F, 27 M**	**5 F, 5 M**	**9 F, 6 M**
Age (years)	38.2	40.0	31.1	35.1
Drinking Days Last Month	18.5	18.9	17.1	-
Standardized Drinks Last Week	27.1	29.6	16.9	-
Drinking Days Last Week	3.6	3.8	2.9	-
Drinks/Drinking Day	6.6	7.2	4.5	-
Heavy Drinking Days Last Week	2.2	2.4	1.6	-
Age at Onset of Drinking Disorder	19.5	19.6	19.2	-
Years Drinking	18.5	20.1	11.9	-

*AA*, alcohol abuse; *AD*, alcohol dependence, *AUD*, alcohol use disorder; *F*, female; *M*, male.

**Table 4:** Variables significantly different between AUD and control subjects

**Variable**		**AUD ave**	**Control ave**	**Fold Change**	**Nominal P**	**BH FDR**
*Medical/Demographic*						
Income		2.4	5.1	-2.16	0.00003	0.013
Years Education		13.2	15.7	-1.19	0.00007	0.015
Blood Pressure (Systolic)		135.2	118.9	1.137	0.00364	0.070
*Neuropsychological*						
Full Scale IQ Percentile		53.3	83.6	-1.57	0.00024	0.025
Full Scale IQ		101.3	118.0	-1.16	0.00025	0.018
Verbal IQ		100.8	116.9	-1.16	0.00038	0.023
Verbal IQ Percentile		52.3	81.6	-1.56	0.00045	0.021
Combined Number-Sequencing Letter-Sequencing Score		10.0	13.1	-1.30	0.00058	0.025
Performance IQ		101.4	115.7	-1.14	0.00108	0.042
Performance IQ Percentile		53.6	80.5	-1.50	0.00115	0.041
General Memory Percentile		51.8	80.0	-1.55	0.00146	0.048
Auditory Immediate Memory Percentile		47.2	76.2	-1.61	0.00151	0.046
Number Sequencing Score		9.2	12.1	-1.32	0.00176	0.046
Visual Delayed Memory Percentile		49.4	73.9	-1.49	0.00249	0.062
Auditory Delayed Memory Percentile		51.0	77.2	-1.51	0.00335	0.079
Letter Fluency Score		10.0	12.9	-1.29	0.00416	0.076
Category Fluency Score		10.9	13.7	-1.26	0.00499	0.088
Letter Sequencing Score		9.8	12.3	-1.25	0.00517	0.087
*Neuroimaging*						
Right Hemisphere Temporal Superiolateral Gyrus		4674.4	5642.7	-1.21	0.00009	0.013
Left Hemisphere Broca’s Area 45		6164.8	7197.7	-1.17	0.00025	0.021
Left Hemisphere Frontal Inferior Triangular Gyrus		2640.2	3232.1	-1.22	0.00042	0.022
Right Hemisphere Pars Opercularis		3956.7	4676.1	-1.18	0.00168	0.047
Right Hemisphere Superiortemporal		11098.8	12513.5	-1.13	0.00336	0.075
Left Hemisphere Parietal Inferior Supramarginal Gyrus		6301.8	7368.1	-1.17	0.00350	0.074
Left Hemisphere Pars Triangularis		3537.5	4218.3	-1.19	0.00351	0.071
Right Hemisphere Frontal Inferior Opercular Gyrus		3034.4	3493.7	-1.15	0.00540	0.088
*Genes*						
HUS1 checkpoint homolog (S. pombe)		493.7	558.3	-1.13	0.05221	-
TP53 tumor protein p53		142.7	217.5	-1.52	0.01665	-
MYC v-myc myelocytomatosis viral oncogene homolog (avian)		216.0	312.4	-1.45	0.01402	-
MUTYH mutY homolog (E. coli)		54.5	68.0	-1.25	0.02417	-
CDK4 cyclin-dependent kinase 4		389.8	446.3	-1.14	0.02410	-
ERCC1 excision repair cross-complementing rodent repair deficiency, complementation group 1		627.9	735.3	-1.17	0.03782	-
MCM5 minichromosome maintenance complex component 5		307.5	396.8	-1.29	0.02045	-

Variables are grouped by category. Benjamini-Hochberg False Discovery Rate (BH FDR) was used to correct P values, except for gene data, where nominal P values are listed.

#### Neuropsychological findings

Compared with controls, AUD subjects demonstrated a trend for decreased scores on auditory and visual memory, verbal and performance IQ, and verbal and performance-based measures of executive function (Table 4). Notably, however, the AUD subjects were not different from the mean population normative values for these standardized testing measures. Rather, the non-drinking controls tended to display above average memory, IQ and executive function.

#### Neuroimaging findings

Of the more than 360 cortical and subcortical brain structures that were examined for potential differences between AUD and control subjects, we observed significant differences in only 8 after correcting for multiple testing (Table 4). These 8 areas comprise 4 distinct brain regions: (1) the left frontal operculum (including Broca’s area 45, inferior triangular gyrus, and pars triangularis); (2) the right frontal operculum (including pars opercularis and frontal inferior opercular gyrus); (3) the right superior temporal area (including the temporal superiolateral gyrus and superiortemporal cortex); and (4) the left parietal inferior supramarginal gyrus (Table 4). All of these brain regions showed significant volume reductions in AUD subjects relative to non-drinking controls, ranging from 1.13 fold to 1.21 fold.

#### Genes

Of the 34 genes examined for expression changes in the human PBLs, we observed nominally significant changes in a total of 7 (*Hus1*, *Tp53*, *Myc*, *Mutyh*, *Cdk4*, *Cdk4*, *Ercc1*, and *Mcm5*; Table 4). All 7 of these genes were decreased in expression in human AUD subjects, and 2 (*Hus1, Mutyh*) overlapped with the genes significantly decreased in the human lymphoblasts. None of the remaining genes overlapped with the genes independently confirmed as changed in the rat QuantiGene Plex assays.

#### Principal components factor analysis

**Figure 3:**
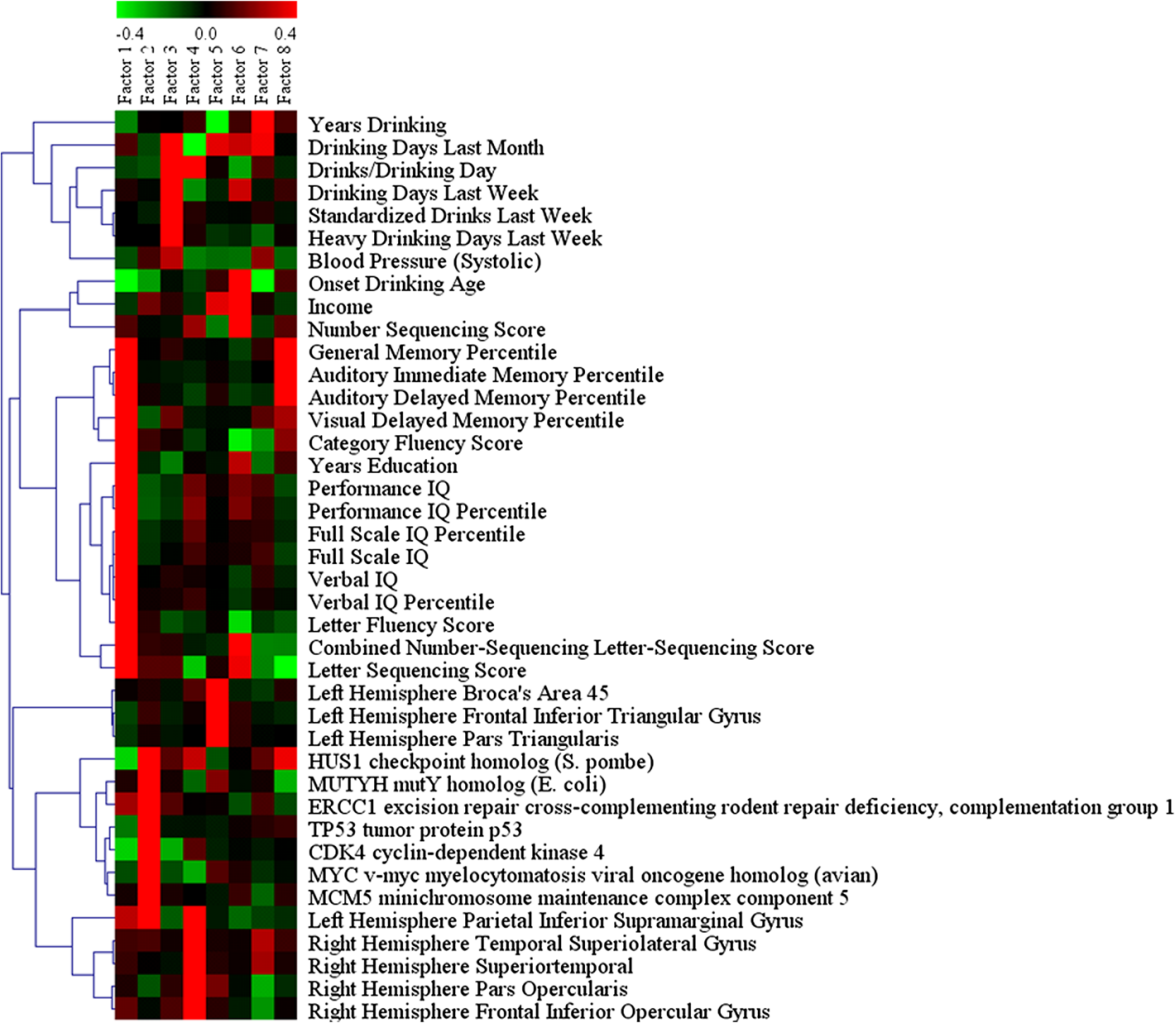
**Hierarchical cluster analysis of the factor loading scores from 40 demographic, medical, neuropsychological, neuroimaging, and gene expression variables that distinguish subjects with alcohol use disorders from healthy controls.** Note the trends for significantly changed neuropsychological variables to load most heavily on factor 1, significantly changed genes to load on factor 2, drinking variables to load on factor 3, and neuroimaging variables to load on factors 4 and 5. One exception to this is the left hemisphere parietal inferior supramarginal gyrus, which showed a strong relationship to the significantly changed gene cluster.

We constructed a correlation matrix for the 40 demographic, medical, neuropsychological, neuroimaging, and gene expression variables that distinguished our AUD and control subjects, followed by a PCA and hierarchical clustering of the loadings for the first 8 factors (which accounted for 75% of the variance in the significantly changed variables). These analyses revealed clear associations in the data for effects on neuropsychological variables (heaviest loading on factor 1), mRNA (heaviest load on factor 2), drinking variables (heaviest load on factor 3), and neuroimaging variables (heaviest load on factors 4 and 5). One exception to this general trend concerned the left hemisphere parietal inferior supramarginal gyrus, which showed a stronger relationship to the significantly changed mRNA cluster that loaded most heavily on factor 2 than to factors 4 and 5, that best accounted for the variance of other neuroimaging measures (Figure 3).

**Figure 4:**
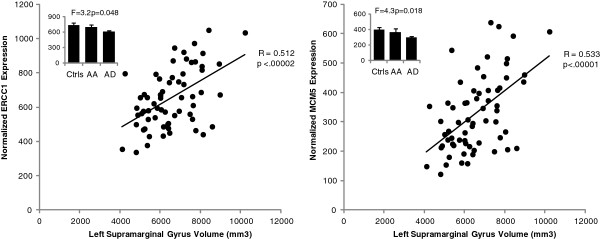
**Highly significant correlations between *Ercc1* (left) or *Mcm5* (right) expression in human PBLs and the volume of the left parietal inferior supramarginal gyrus.** The histogram plots (inset) show the results of an ANOVA comparing the expression levels of *Ercc1* and *Mcm5* in all three subject groups. Note that both genes showed a significant main effect of diagnosis, but there was a clear trend for alcohol dependent (AD) subjects to show more of a decrease in expression compared to controls (Ctrls) than alcohol abusing (AA) subjects.

To further probe the PCA results specifically in regards to the gene expression results, we examined the Bonferroni-corrected correlation matrix and identified two striking findings. First, all 7 of the significantly changed genes showed robust positive correlations with each other, almost all of which survived Bonferroni correction (not shown). Secondly, the expression of both *Ercc1* and *Mcm5* showed highly significant correlations with the volume of the left hemisphere parietal inferior supramarginal gyrus (Figure 4). This relationship was suggested by the close clustering of these variables in the PCA matrix (Figure 3). Further examination of the expression levels of these genes using a one way ANOVA indicated significant differences across the 3 diagnostic groups (AA, AD and Controls) with a clear trend for AA subjects to show only modest decreases (which were not significant) while AD subjects showed significant decreases relative to controls (Figure 4, inset graphs).

Three other groups of nominally-significant correlations were also noted for *Ercc1* and *Mcm5*, including: (1) positive correlations with multiple brain region volumes, including the right hemisphere equivalent of the left parietal inferior supramarginal gyrus (r = 0.54), the left and right middle temporal lobes (r = 0.33 to 0.44), the left and right caudate nucleus and thalamus (r = 0.30 to 0.47), and total left and right cortical gray matter (r = 0.37 to 0.41). (2) positive correlations (r = 0.25 to 0.39) with 13 of the 15 significantly changed neuropsychological variables listed in Table 4 (the only exceptions being the Number Sequencing Score and Visual Delayed Memory Percentile); and (3) positive correlations between *Ercc1* and absolute eosinophil and basophil counts along with a modest negative correlation between *Ercc1* levels and absolute lymphocyte counts (r = -0.30).

## Discussion

The effects of alcohol use disorders exert a tremendous impact on the central nervous system of adult humans [1,2,17]. Alcohol exposure affects the normal processes of neuronal proliferation, repair, and apoptosis in humans [10,18] and in animal models [19,20]. The p53-signaling pathway regulates each of these processes [21] and has been implicated in ethanol-induced changes in the central nervous system [15,22]. The current study focuses on examination of the expression of p53-related genes in human and rat PBLs and identifies sets of ethanol-responsive genes that are correlated with changes in brain structure and function.

One of our major findings is that the expression of the p53 gene and six p53-related genes are decreased in the blood of human subjects with AUD. Furthermore the expression levels of several of these genes (particularly *Ercc1*, *Mcm5*, and *p53*) are positively correlated with the volumes of several cortical and subcortical brain regions, (particularly the left parietal supramarginal gyrus) as well as several measures of significantly affected neuropsychological variables. When combined with our results from the mouse NSCs and rat PBLs, this suggests that alterations in p53 signaling may be occurring within the brain of subjects with AUD. Support for this possibility comes from studies of rat pups exposed to ethanol, which demonstrated reduced *p53* expression in the developing cortex [22], as well as gene expression studies of postmortem human brain. Specifically, one study of the frontal cortex of adult human alcoholics identified changes in 35 neurogenesis-related transcripts and 11 apoptosis-related transcripts – both of which are highly regulated by p53 [9].

Our findings regarding *Ercc1* levels are particularly noteworthy for several reasons. The *Ercc1* gene-product is involved in nucleotide excision repair (NER) of damaged DNA [23]. This process is potentially highly relevant to understanding the effects of ethanol on genomic integrity. For example, the process of NER is a critical means for removing harmful DNA adducts that can form after exposure to ethanol or its metabolites. Indeed, in cancer therapy, NER is viewed as the primary mechanism whereby platinum-DNA adducts are removed from the DNA of tumor cells following cisplatin treatment. Accordingly, *Ercc1* activity levels have been viewed as a potentially important prognostic biomarker for tumor responsiveness to cisplastin [24,25]. In AUD subjects *Ercc1* expression was nominally correlated with the age of onset of the alcohol use disorder and robustly and significantly correlated with the volume of the supramarginal gyrus in the left inferior parietal lobe region. Thus, the importance of *Ercc1* in repairing ethanol-associated DNA damage may increase over the lifespan - particularly for this brain region, which is now well-established as involved in several aspects of language processing (e.g., [26]). Several recent studies lend even stronger support for this notion. For example, the *Ercc1* null mouse has been used as a model of accelerated aging (progeria) because it exhibits shunted growth, wasting, ataxia, and premature death by 1-2 months of age [27,28]. Mice with reduced expression of *Ercc1*, however, show a less rapid progeria phenotype, surviving until 4-6 months of age, but exhibit clear signs of metabolic, neurologic, and cognitive decline (including learning and memory deficits) along with neurodegenerative changes (elevated expression of markers that indicate reactive astrocytosis and apoptosis) [29,30]. A recent follow up study of these mice by Vegh and colleagues [31] revealed that even before these types of changes are seen in the hippocampus, there are significant reductions in the expression levels of numerous proteins involved in synaptic function during the early stages of accelerated aging due to reductions in *Ercc1* expression, which they proposed were the cause of the learning and memory deficits in these mice. Thus, DNA damage repair processes may be critical for preventing age-dependent cognitive decline in otherwise normal mice. Given that our AUD subjects showed significantly reduced *Ercc1* expression and performance on several standardized measures of verbal function, the present study strongly suggests a role for DNA repair processes and *Ercc1* in preventing alcohol-dependent cognitive decline as well. Clearly, the relationship between ethanol consumption, *Ercc1* expression, brain volume, neuropsychological function, aging, and cancer risk merits further investigation. In addition, there may be other explanations for the changes in *Ercc1* levels that we have not considered.

Our findings regarding *Mcm5* are also highly novel and may relate to the *Ercc1* findings in terms of the relationship with the volume of the left inferior parietal supramarginal gyrus. The *Mcm5* gene product is a chromatin-binding protein that regulates the initiation of the cell cycle at the G_0_-G_1_/S transition [32] and is negatively regulated by *p53*[33]. Not only is *MCM5* down-regulated in human PBLs, it is also down-regulated in the frontal cortex of human alcoholics [8,9] and in mouse neural stem cells exposed to ethanol (11-fold decrease; 15). Such changes could lead to a reduction in cell proliferation. If such effects are also present in the brain of subjects exposed to ethanol during sensitive developmental periods, this could help explain why alcohol-induced decreases in *Mcm5* are correlated with reductions in the volumes of several brain regions, such as the left inferior parietal supramarginal gyrus of AUD subjects.

Together, the *Tp53*, *Ercc1*, and *Mcm5* genes represent intertwined pathways altered by ethanol exposure. Alcohol-induced down-regulation of *Mcm5* and other cell cycle-related transcripts (*Myc*, *Cdk4*, *Pttg1*) may lead to checkpoint restriction at both G_0_ and G_2_/M. This interruption of proliferation is necessary for activation of DNA-repair mechanisms (i.e. *Hus1*, *Mutyh*, *Ercc1*) secondary to alcohol-induced oxidative stress [34]. The master regulator of these processes is *Tp53*, a gene that controls cell fate through the orchestration of repair and proliferation and the initiation of apoptosis [35]. We can now confirm that the *p53*-pathway is affected by alcohol in both the CNS and peripheral blood of humans and animal models. Moreover, this study has identified specific players within the *p53*-pathway that serve as biomarkers for the deleterious effects of alcohol. These genes are not only correlated with changes in brain volume and function, but may also play an integral role in the pathophysiology of alcohol-induced CNS damage.

There are a number of notable limitations to the present study that should be mentioned. First, we did not measure the expression of genes in the brains of AUD subjects since this was not a postmortem study. Nonetheless, our results on *p53*-related genes are consistent with results from prior postmortem studies. Second, the significant changes seen in the rat and human PBLs appeared to be largely distinct, although both sets of data clearly supported our hypothesis that *p53*-related genes would be affected, and there was significant association between the rat PBL results and mouse NSC results. Third, we did not assess brain volumes or brain function in the rat drinking model, and thus far we have only established the relationship between changes in *p53*-related genes in a single drinking paradigm. Finally, it is possible that some of the differences we are ascribing to ethanol that were found between AUD and control subjects may be influenced by the fact that our controls appeared to be very high functioning relative to the general population on several neuropsychological measures. Although this potential bias may be difficult to avoid when selecting non-drinking subjects for inclusion in this type of study, it also argues in favor of additional studies.

## Conclusion

We have identified a specific set of *p53*-related genes that are altered by ethanol exposure in both humans and animal models in proliferating cells of the CNS and blood. Though the expression changes of these genes are important in understanding the mechanisms underlying ethanol-induced CNS damage, their expression level changes in peripheral blood are perhaps equally important as biomarkers of CNS damage. For example, the expression of *Mcm5* and *Ercc1* are correlated with increased alcohol consumption and decreased volume of the thalamus and caudate bilaterally. Similarly, *Mcm5* and *Ercc1* demonstrate robust correlations with category fluency and verbal IQ, respectively. Because *Mcm5* and *Ercc1* are strongly correlated with a cluster of *p53*-related transcripts this entire group of genes may hold clinical value as biomarkers of CNS damage and functional impairment in patients with AUD.

## Competing interests

None of the authors have any competing interests to report for this study.

## Authors’ contributions

FAM, SDH and ZSM designed the study. FAM and SDH took the primary role in data analysis. SDH, LL, JR, PB, ES, and KG performed various lab assays or assisted with data analysis. LL, PB, YAM, NA, KC, ZSM, and FAM recruited and evaluated the human subjects. SDH, LL, KG, and FAM wrote different portions of the manuscript. All authors read and approved the final manuscript.

## Supplementary Material

Additional file 1 **Figure S1. **Experimental overview. The rationale for the present study was based on our recently published findings [15,16] regarding the *in vitro* effects of ethanol on mRNA expression in mouse neural stem cells (NSCs), where changes in a considerable portion of genes involved in p53 signaling, cell cycle regulation, apoptosis and DNA damage and repair were observed and independently confirmed using real-time quantitative RT-PCR (qRT-PCR). In the present study, we first examined whether there were similar changes in peripheral blood leukocytes (PBLs) in an *in vivo* rat binge drinking model using microarray data. Then, based on the considerable overlap and correlated changes, we confirmed several of the rat findings and tested their validity in two sets of human samples: PBLs from subjects with alcohol use disorders, and lymphoblasts (LBs) from a normal human subject. We noted that all four of these data sources showed evidence of dysregulation of the genes of interest, although the specific genes most affected could vary across models. In addition, several of the genes showed highly significant correlations with medical, neuropsychological, neuroimaging, and demographic traits in our human subjects. Click here for file
