# Supplementary material for: Evaluation of cell proliferation, apoptosis, and dna-repair genes as potential biomarkers for ethanol-induced cns alterations
Source: BMC Neurosci. 2012 Oct 25;13:128. doi: 10.1186/1471-2202-13-128 (PMC3519626; doi:10.1186/1471-2202-13-128)

EXPERIMENTAL WORKFLOW

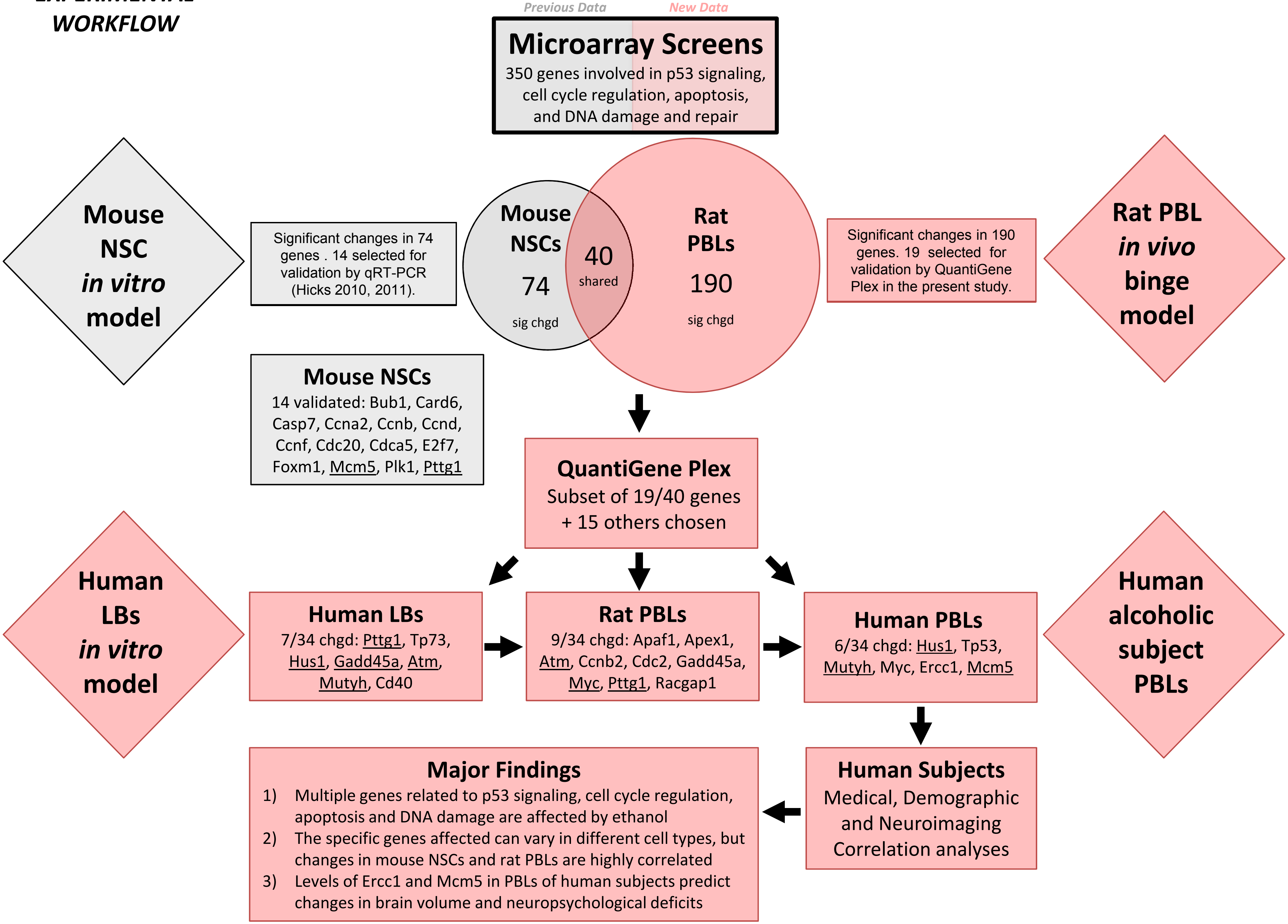

Supplement: Additional file 1 — Figure S1. Experimental overview. The rationale for the present study was based on our recently published findings [15,16] regarding the in vitro effects of ethanol on mRNA expression in mouse neural stem cells (NSCs), where changes in a considerable portion of genes involved in p53 signaling, cell cycle regulation, apoptosis and DNA damage and repair were observed and independently confirmed using real-time quantitative RT-PCR (qRT-PCR). In the present study, we first examined whether there were similar changes in peripheral blood leukocytes (PBLs) in an in vivo rat binge drinking model using microarray data. Then, based on the considerable overlap and correlated changes, we confirmed several of the rat findings and tested their validity in two sets of human samples: PBLs from subjects with alcohol use disorders, and lymphoblasts (LBs) from a normal human subject. We noted that all four of these data sources showed evidence of dysregulation of the genes of interest, although the specific genes most affected could vary across models. In addition, several of the genes showed highly significant correlations with medical, neuropsychological, neuroimaging, and demographic traits in our human subjects. [file 1471-2202-13-128-S1.pdf]
